# Preservation of tumor-host immune interactions with luciferase-tagged imaging in a murine model of ovarian cancer

**DOI:** 10.1186/s40425-015-0060-6

**Published:** 2015-05-19

**Authors:** John B Liao, Kelsie J Ovenell, Erin E M Curtis, Denise L Cecil, Marlese R Koehnlein, Lauren R Rastetter, Ekram A Gad, Mary L Disis

**Affiliations:** Division of Gynecologic Oncology, Department of Obstetrics and Gynecology, University of Washington, 1959 NE Pacific St., Seattle, WA 98195 USA; Tumor Vaccine Group, Center for Translational Medicine in Women’s Health, University of Washington, 850 Republican St., Seattle, WA 98109 USA; Swarthmore College, 500 College Ave, Swarthmore, PA 19081 USA

**Keywords:** Ovarian cancer, Mouse models, Immune therapies

## Abstract

**Background:**

Ovarian cancer is immunogenic and residual tumor volume after surgery is known to be prognostic. Ovarian cancer often follows a recurring-remitting course and microscopic disease states may present ideal opportunities for immune therapies. We sought to establish the immune profile of a murine model of ovarian cancer that allows in vivo tumor imaging and the quantitation of microscopic disease.

**Results and Discussion:**

Baseline imaging and weight measurements were taken within 1 and 2 weeks after intraperitoneal tumor injection, respectively. Significantly higher photons per second from baseline imaging were first observed 5 weeks after tumor cell injection (p < 0.05) and continued to be significant through 8 weeks after injection (p < 0.01), whereas a significant increase in weight above baseline was not observed until day 56 (p < 0.0001). Expression of luc2 in ID8 cells did not alter the cellular immune microenvironment of the tumor. FOXP3+ T cells were more likely to be detected in the intraepithelial compartment and CD4+ T cells in the stroma as compared to CD3+ T cells, which were found equally in stroma and intraepithelial compartments.

**Conclusions:**

Use of an intraperitoneal tumor expressing a codon-optimized firefly luciferase in an immunocompetent mouse model allows tumor quantitation in vivo and detection of microscopic tumor burdens. Expression of this foreign protein does not significantly effect tumor engraftment or the immune microenvironment of the ID8 cells in vivo and may allow novel immunotherapies to be assessed in a murine model for their translational potential to ovarian cancers in remission or minimal disease after primary cytoreductive surgery or chemotherapy.

**Methods:**

Mouse ovarian surface epithelial cells from C57BL6 mice transformed after serial passage in vitro were transduced with a lentiviral vector expressing a codon optimized firefly luciferase (luc2). Cell lines were selected and luc2 expression functionally confirmed in vitro. Cell lines were intraperitoneally (IP) implanted in albino C57BL/6/BrdCrHsd-Tyrc mice and albino B6(Cg)-Tyrc-2 J/J mice for serial imaging. D-luciferin substrate was injected IP and tumors were serially imaged in vivo using a Xenogen IVIS. Tumor take, weights, and luminescent intensities were measured. Immunohistochemistry was performed on tumors and assessed for immune infiltrates in stromal and intraepithelial compartments.

**Electronic supplementary material:**

The online version of this article (doi:10.1186/s40425-015-0060-6) contains supplementary material, which is available to authorized users.

## Background

The ability of the immune system to recognize and respond to ovarian cancers has been known to be important in prognosis [1,2]. This observation has sparked much interest in applying immune therapies and vaccines for the treatment of this malignancy, however, the promise of immune therapy has yet to be realized in ovarian cancer patients despite a number of approaches taken to model the disease in mice. Mouse models for ovarian cancer may have more success in translational oncology if they can replicate the immune microenvironment and allow quantitation of low tumor volumes, two factors that are known to impact outcomes in human disease. The presence of intratumoral T cells in ovarian cancer is an independent prognostic factor for progression free survival (PFS) and overall survival (OS) by multivariate analysis and underscores the specific importance of T-cells [1-7]. Regulatory T cells, another subset of T cells that can modulate immune responses and maintain tolerance to self-antigen, have been shown to predict poor patient survival in ovarian cancer [2,8]. For advanced ovarian cancers, tumor volume continues to be an important factor in prognosis, where patients who achieve microscopic residual have a 34 months median overall survival advantage over those who have even 0.1 cm macroscopic disease after cytoreductive surgery [9].

Some of the earliest attempts to model ovarian cancer in mice involved the implantation of human tumor tissue, subcutaneously, in immunodeficient mice [10]. This allowed the direct measurement of external tumors but did not replicate the location or important immune interactions. More recently, human tumor xenografts have been successfully implanted orthotopically in NOD-scid immunodeficient mice [11-13]. The development of effective mouse models to study ovarian cancer has also been hampered by an incomplete understanding of the molecular events that lead to carcinogenesis. A number of genetically induced murine models have been developed that exploit mutations that lead to loss of expression of tumor suppressor genes and overexpression of oncogenes, individually and in combination, through the use of conditional deletion and expression techniques directed to the murine reproductive tract [14-16]. Tissue specific promoters have also been utilized to focus expression of oncogenes in the murine ovary during development, but these efforts were insufficient to transform ovarian surface epithelium, inhibited reproductive function, and/or introduced oncoproteins such as T antigen, which have no known role in ovarian carcinogenesis [17-19]. While a sequence of molecular and cellular events has been shown to lead to tumor progression in syngeneic mouse models of ovarian cancer [20], even high grade serous ovarian cancers in humans exhibit a great degree of heterogeneity, and carcinogenesis cannot yet be attributed a defined sequence of mutational events[21], so it is unclear how closely genetically induced murine models would replicate human disease. Application of chemical carcinogens such as 7,12-dimethylbenzanthracene (DMBA) have been used to induce cancers of the reproductive tract in mice with very low efficiency [22]. When these efforts were attempted in rats, success rates increased to 50%, but it also induced epithelial cancers of the endometrium and cervix [23]. However, chemical carcinogens have yet to be definitively associated with human ovarian carcinogenesis. Mouse ovarian surface epithelial cells undergo transformation after serial passages *in vitro* and have represented a syngeneic and immunocompetent mouse model [24]. The intraperitoneal location of these more recent approaches to modeling ovarian cancer in mice raises the same issues seen human ovarian cancer: tumor quantitation and detection of low volume disease.

Murine ovarian tumors have been previously imaged *in vivo* using luciferase [25-27]. We sought to evaluate this approach when it is enhanced to use a codon-optimized protein and mutant mouse strains that permit improved transmission of light from intraperitoneal tumors. Use of these modifications has been reported to allow detection *in vivo* to the level of 10 cells in albino mice [28]. It is not known whether the optimized expression of a xeno-antigen or use of mutant C57BL6 mice will alter tumor engraftment of this mouse model or how quantitation of these tumors will track with external measures. It is also unknown whether the expression of xeno-antigen will alter the intraperitoneal tumor microenvironment potentially eliciting a shift from immunosuppressive to inflammatory.

## Materials and Methods

### Lentiviral infection of ID8 with luciferase vector and cell line selection

ID8 cells, ovarian surface epithelial cells derived from the C57B6 mice (obtained from K. Roby, University of Kansas) [24], were plated at 3x10^5^ cells per well (6-well plate; Corning, Inc.) and incubated overnight at 37°C/5% CO_2_. Media consisted of Dulbecco’s Modification of Eagle’s Medium w/L-glutamine (DMEM; Corning Inc.), 4% fetal bovine serum (FBS; Gemini), 0.09 mg/ml penicillin-streptomycin (Corning, Inc.), and 1× insulin/transferrin/selenium (ITS; Gibco). Cells were infected with 2 mL/well pLentiIII-Luc2 viral vector supernatant (Applied Biological Materials Inc.) in the presence of 8 μg/ml polybrene (EMD Millipore Corporation). After overnight incubation at 37°C/5% CO_2_, the viral supernatant and media with polybrene were removed and the plate was washed with PBS prior to the addition of warmed media. Cells were cultured in growth media for 72 hours and then placed under drug selection with 1 μg/mL puromycin, added daily (Invitrogen). Colonies were selected using 3 mm cloning disks soaked in 0.25% trypsin-EDTA (Invitrogen) and grown to confluence in 6 well plates. Cells were then trypsinized, spun, and suspended to a concentration of 5×10^4^ cells/100 μl. One hundred μl of cells were added per well in a white 96-well plate (EMD Millipore Corporation) and equal volume of 3000 μg/ml d-luciferin was added immediately before reading. Lines were selected based maximal relative light units after addition of substrate as a measure of functional luciferase expression (Additional file 1: Figure S1).

### Mice

C57/BL/6/BrdCsHsd-Tyr^c^ (Harlan Laboratories) and B6(Cg)-Tyrc-2 J/J mice (Jackson Laboratories) were acquired and maintained under standard pathogen-free conditions at the University of Washington. Both C57/BL/6/BrdCsHsd-Tyr^c^ and B6(Cg)-Tyrc-2 J/J mice contain a mutation in the c(tyrosinase) gene yielding an albino coat and have the H-2^b^ immune haplotype. 6–8 week old female mice were used for this study and allowed to acclimate for one week in-house prior to treatment. Blood was collected by orbital bleed, every two weeks throughout all studies. Mice were directly observed and weighed every 2–3 days after tumor growth was evident. Mice were euthanized when they exhibited clinical signs of disease or distress (i.e. cachexia, anorexia, or increased respiratory rates), development of ascites or when tumors began to interfere with normal bodily functions (i.e. ambulation, eating, drinking, defecating, and urinating). All protocols were approved by the Institutional Animal Care and Use Committee.

### In vivo propagation of ID8-luc2 tumors

Mice were given a 200 μL intraperitoneal injection of ID8 cells, ranging from one to five million cells per mouse. With the mouse in the supine position, half the dose was injected using a 25 gauge needle in the lower left quadrant and the other half in the lower right quadrant. At designated intervals after tumor implant, the mice were imaged to monitor tumor progression. The two mouse strains were evaluated with either a low-load (1×10^6^ cells) or a high-load (5×10^6^ cells) of either a low (greater than 10,000 relative light units) or high (greater than 50,000 relative light units) expression luc2 line. A total of 32 mice were injected with tumors, at 4 mice per group, as follows for both mouse strains: low-load + low-expression line, low-load + high-expression line, high-load + low-expression line, and high-load + high-expression to select a condition that gave a significant change in total luminescent flux at 4 weeks (Additional file 2: Figure S2). All subsequent studies were performed using 5 mice per treatment group with Harlan C57BL/6 mice with the high expression luc2 line at 5×10^6^ cells/mouse. Significance was determined using repeated measures ANOVA for weights. All time points were compared to the earliest time point of 14 days or 2 weeks after tumor cell injection.

### Bioluminescence/Imaging

Bioluminescent images were taken with Xenogen IVIS using D-luciferin, (In Vivo Imaging Solutions) as previously described [26]. Images were normalized using Living Image software (PerkinElmer) with a minimum and maximum radiance of 5×10^5^ and 2.5×10^6^ photons/sec, respectively. Maximum luminescent intensity and total flux in photons per second were calculated and reported for each mouse’s abdominal region in photons/sec. Significance was determined using one way Anova for luminescence. All time points were compared to the earliest time point of 14d or 2 weeks after tumor cell injection. Successful engraftment of intraperitoneal tumors was defined as 5×10^5^ photons/sec. This value was based on prior studies which demonstrated that failure to achieve more than 10^5^ photons/sec bioluminescent emission correlated with rejection of implanted tumor [29].

### Enzyme-linked immunosorbent assay

Blood samples were centrifuged and sera collected for analysis. Serum antibodies to luciferase were assessed using an indirect ELISA [30]. Alternate columns on Immulux HB plates (Dynex Technologies, Inc.) were coated with 5 μg/mL firefly luciferase (Abcam) in carbonate buffer, determined optimal by a checkerboard titration. Serially diluted, purified human IgG (Sigma) provided a standard curve. Plates were incubated overnight at 4°C followed by a one hour block with 1X PBS/5% bovine serum albumin (BSA; Fitzgerald Industries International) at room temperature. After washing three times with ELISA wash buffer: 1XPBS/ 0.1% Tween (Thermo Fisher Scientific Inc), serum samples obtained by orbital bleeding from mice with tumors expressing luc2 were diluted 1:100 in 1XPBS/1% BSA, and incubated for an hour. A positive control of rabbit anti-firefly luciferase 36 μL diluted in 414 μL (Abcam) and negative control of normal mouse serum 10 uL diluted in 115 μL 1XPBS/1% BSA were run on every plate. Plates were re-run if positive control value less than 20 fold of the negative control value as well as if the negative controls were more than 5% above background. Plates were washed three times with wash buffer, then 1:100,000 secondary antibody of goat anti human, mouse or rabbit-IgG-horseradish peroxidase (HRP; SigmaAldrich) conjugates was added to control, standard curve, and experimental wells and incubated for 45 minutes. After washing again, plates were developed with TMB (KPL) and the reaction was read at an optical density of 640 nm until the well containing the standard concentration of 0.078 μg/mL reached 0.3 OD. The reaction was stopped with an equal volume of 1 N HCL and read at 450 nm. The OD_450_ of each serum dilution was calculated as the OD_450_ of antigen-coated well minus the OD_450_ of carbonate buffer coated wells. Values in μg/mL for each OD_450_ were calculated from the log-log equation of the line for the standard curve on each plate as plotted by Softmax Pro 5.3 (Molecular Devices Corp). A sample was defined as positive if the value was greater than 2 standard deviations above the mean value of controls, or mice implanted with the parental ID8 cell line (n = 10). Intra-assay and inter-assay coefficients of variation were 8% and 12.5% respectively (8 plates evaluated).

### Immunohistochemistry

Staining was performed on a Leica Bond Automated Immunostainer. Frozen sections were chilled in acetone and washed in PBS prior to antigen retrieval at 100°C for 10–20 minutes. Slides were then blocked with 10% normal goat sera in TBS for 10 minutes followed by incubation with Rat anti-mouse CD3 (AbD Serotec, Cat. No. MCA1477, 1.0 mg/mL) at 1:500 dilution, anti-mouse FOXP3 (eBioscience, Cat. No. 14–5773, 0.5 mg/ml) 1:250, anti-mouse CD4 (BD Pharmingen, Cat. No. 550278, 62.5 μg/ml) 1:500, or rat IgG isotype control (BD Pharmingen, Cat No. 553986, Lot No. MO53508, 0.5 mg/mL) 1:500 dilution in Bond primary antibody diluent (Leica) for 30 minutes at room(Leica) for 30 minutes at room temperature. Secondary antibody, rabbit anti-rat IgG (Vector Laboratories Inc., Cat No AI-4001, 0.5 mg/mL; diluted 1:300 in 5% NGS/1XTBS) was incubated for 8 minutes at room temperature. Sections were then incubated with 8 μg/mL goat anti rabbit poly-HRP polymer secondary detection (Leica) for 8 minutes at room temperature, followed by Leica Bond Mixed Refine DAB substrate detection for 10 minutes at room temperature (Leica). After washing with diH_2_O the sections were counter stained with Mayer hematoxylin solution (Newcomer Supply, Cat No. 12013) dehydrated through 100%, cleared in xylene and mounted with synthetic resin mounting medium and #1.5 coverslip. Quantification of tumor infiltrating lymphocytes (TILs) was performed as previously described using H&E staining to identify stromal and intraepithelial sections and reported as a percentage of positively stained cells for CD3, CD4, and FOXP3 [2].

### Flow cytometry

Splenocytes and TILs were analyzed by flow cytometry. Lymphocytes from tumor and spleens were isolated as previously described [31,32]. Flow cytometry was performed as shown previously and anti-mouse CD16/CD32 antibody (BD Pharmingen) was used to block nonspecific binding [33]. The following fluorochrome-conjugated antibodies were used in 2×10^6^ cells: 0.4 μg CD45 (eBioscience, clone #30-F11), 0.4 μg CD3 (BD Pharmingen, clone #145-2C11), 0.4 μg CD4 (BioLegend, clone #GK1.5), 0.4 μg CD8 (eBioscience, clone #53-6.7), 0.4 μg CD11b (eBioscience clone #M1/70), 0.4 μg GR-1 (BD Pharmingen clone #RB6-8C5), and 1 μg Foxp3-Alexa488 (eBioscience, clone # FJK.16 s). Stained cells were acquired with FACS Canto flow cytometer (BD Bioscience) and 1×10^6^ to 2×10^6^ cells were analyzed with FlowJo software (Tree Star Inc.). Results are reported as total percentage of a cell population or ratio of cell quantities, as indicated.

### Statistical analysis

Graphs and statistical comparisons were completed using GraphPad Prism v5.04 software. Unpaired t-tests and one-way ANOVA were used. Significance was defined as p < 0.05 for all statistical tests.

## Results

### Bioluminescent imaging is a more sensitive indication of tumor growth than weight gain in ovarian cancer mouse models

**Figure 1:**
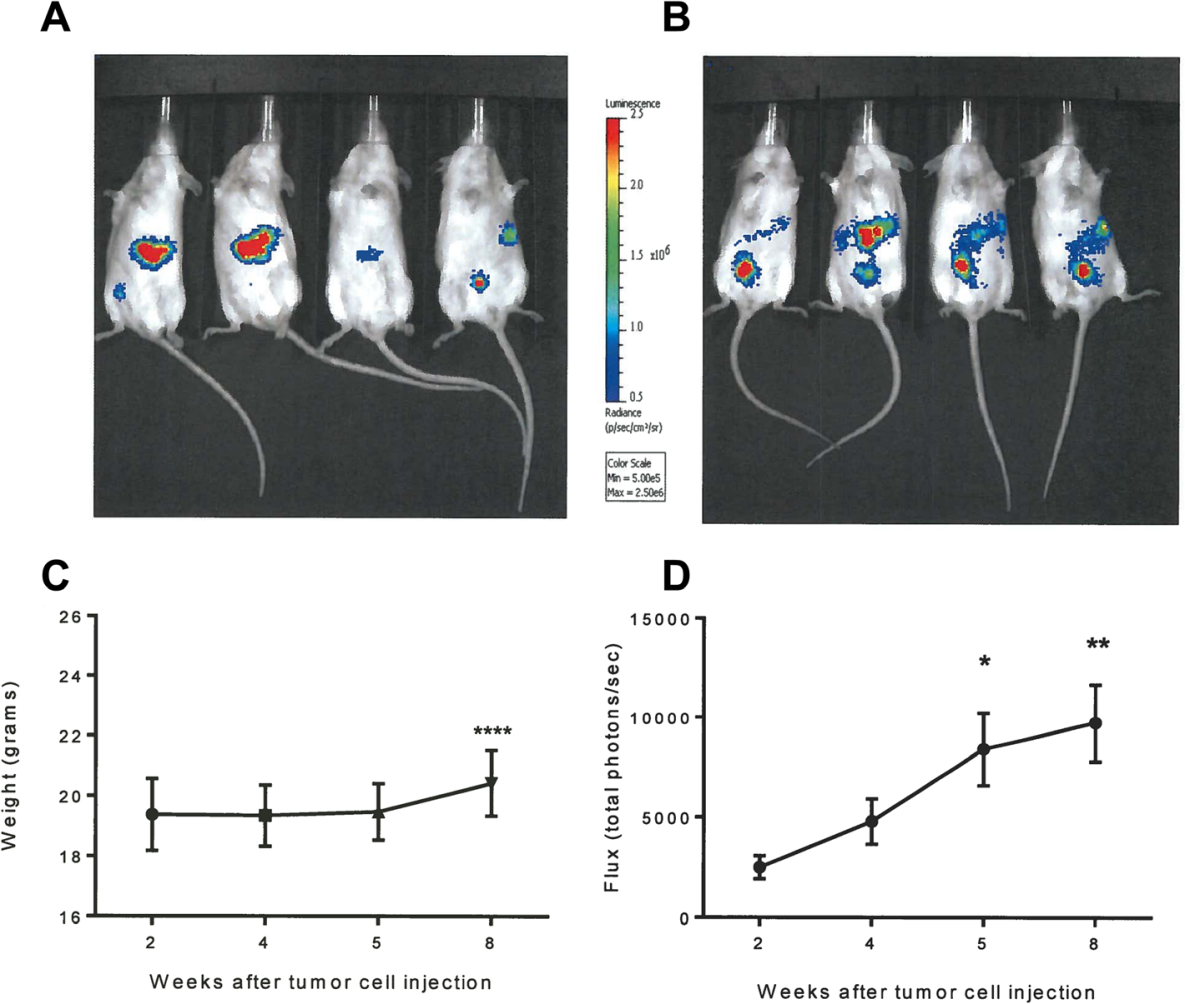
***Bioluminescent imaging detects tumor prior to significant weight change*** **Representative bioluminescent imaging of ID8 Luc2 implant at 2 (A) and 4 weeks (B) after injection.** Mice are placed in the same positions for both images. **(C)** Average weight of mice at 2, 4, 5, and 8 weeks after tumor cell injection (x-axis), n = 32. **(D)** Average luminescent units at 2, 4, 5, and 8 weeks after tumor cell injection (x-axis), n = 32. *p < 0.05, **p < 0.01, ****p < 0.0001.

We wished to examine whether factors such as number of tumor cells injected IP, choice of C57Bl6 mutant strains, and expression level of luc2, a codon-optimized firefly luciferase, in a selected line impacted tumor detection and engraftment rates. We hypothesized that use of bioluminescent imaging would enhance detection of small volume tumors in advance of external measures and that expression of a foreign antigen, luc2, would not impair engraftment. In order to determine if a lower number of implanted tumor cells could still be detectable by bioluminescence and still have a high engraftment rate, we injected mice with IP tumors of the ID8 cells with high expression of luc2 at both the reported number used in the parental line, 5×10^6^ and 1×10^6^ cells. 81% of mice injected with 1×10^6^ cells had detectable tumor by 2 weeks after implantation. All mice injected with 5×10^6^ tumor cells had detectable tumors by bioluminescent imaging 2 weeks after implantation. Serial images showed a distribution of bioluminescent tumor approximating progressive metastatic peritoneal carcinomatosis. Compared to baseline imaging and weight measurements taken within 1 and 2 weeks after intraperitoneal tumor injection, significantly higher photons per second from baseline imaging were first observed 5 weeks after tumor cell injection (p = 0.0144) and continued to be significant through 8 weeks after injection (p = 0.002), whereas a significant increase in weight above baseline was not observed until day 56 (p < 0.0001; Figure 1).

We also examined 2 selected lines of ID8-luc2 cells, a low expression line A (n = 16) and a high expression line B (n = 16) to assess if high levels of overexpression of a foreign protein such as luc2 will effect tumor engraftment. No significant change between the two lines was seen at 2, 4, and 6, weeks, by one way ANOVA (p = 0.16; Additional file 2: Figure S2A). In order to optimize light transmission we also tested the selected ID8-luc2 lines in two C57Bl6 based mouse strains with white coats from 2 vendors. There were no significant differences seen between these groups and tumors were visualized at 2 weeks with both strains (p > 0.05; Additional file 2: Figure S2A).

### Development of endogenous luc2 specific IgG antibodies did not impact tumor growth

**Figure 2:**
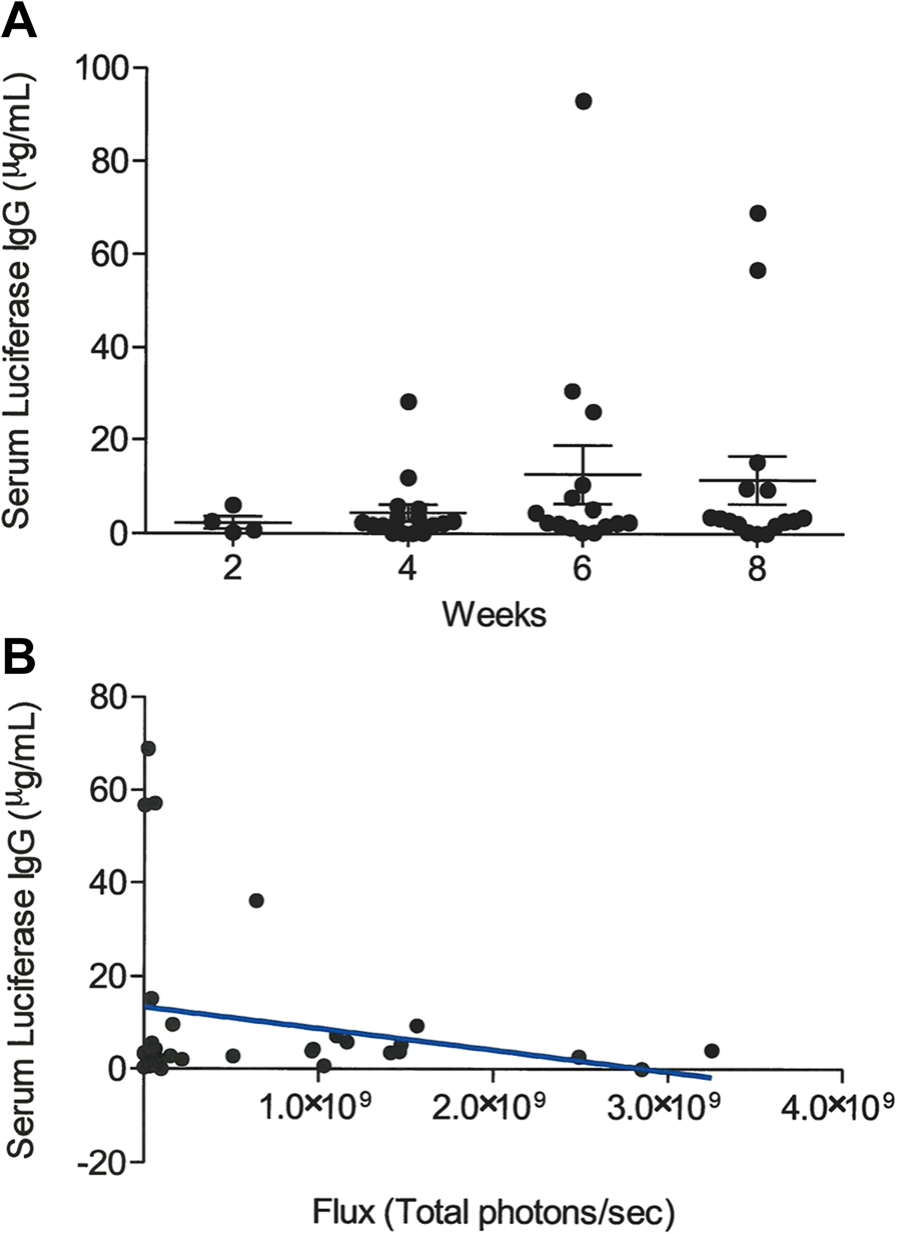
***Development of endogenous luc2 specific IgG antibodies did not impact tumor growth.*** **(A)** Serum firefly luciferase specific IgG μg/mL (y-axis) for ID8 luc2 mice, over time in weeks (x-axis). Bars represent mean and SEM. n = 16 **(B)** Bioluminescent imaging of ID8 Luc2 mice at 4 and 6 weeks post tumor implant. Serum firefly luciferase specific IgG μg/mL (y-axis) compared to matched bioluminescent flux (x-axis). Linear regression line shown in blue. (n = 32).

We hypothesized that expression of a foreign antigen may generate autoantibodies, but that these may not be sufficient to cause tumor regression, since autoantibodies generated against potential tumor associated antigens do not correlate with overall survival in 2 out of 3 antigens studied in ovarian cancer patients [34]. Serum antibodies specific for firefly luciferase were elevated in 3 of 16 (18.75%) tumor bearing mice at 6 weeks after implantation with 5×10^6^ cells, with a mean +/− SEM of 12.6 μg/mL +/− 6.2, (range: 0.17 to 93.1) using the two cell lines and both mouse strains (Figure 2A). The proportion of mice with serum antibodies to luciferase at a concentration greater than the mean and SEM were 2 of 16 (6.25%) on week 4, 3 of 16 (18.75%) on week 6, and 2 of 16 (12.5%) on week 8. These elevated levels were not significantly different by one way ANOVA, showing that development of antibodies did not vary significantly by time of exposure to luciferase (Figure 2A). Linear regression analysis performed on serum antibody levels and total bioluminescent flux did not show a significant correlation or a significantly non-zero slope (R^2^ = 0.05411; p = 0.20; Figure 2B). The higher expressing line induced a positive antibody response at earlier time points, as early as 4 weeks, but serum antibody responses were ultimately detected in both mouse strains, using both high and low expressing lines, and both high and low cell numbers by eight weeks (Additional file 2: Figure 2B).

### Expression of luc2 in ID8 cells does not alter the cellular immune microenvironment of the tumor

**Figure 3:**
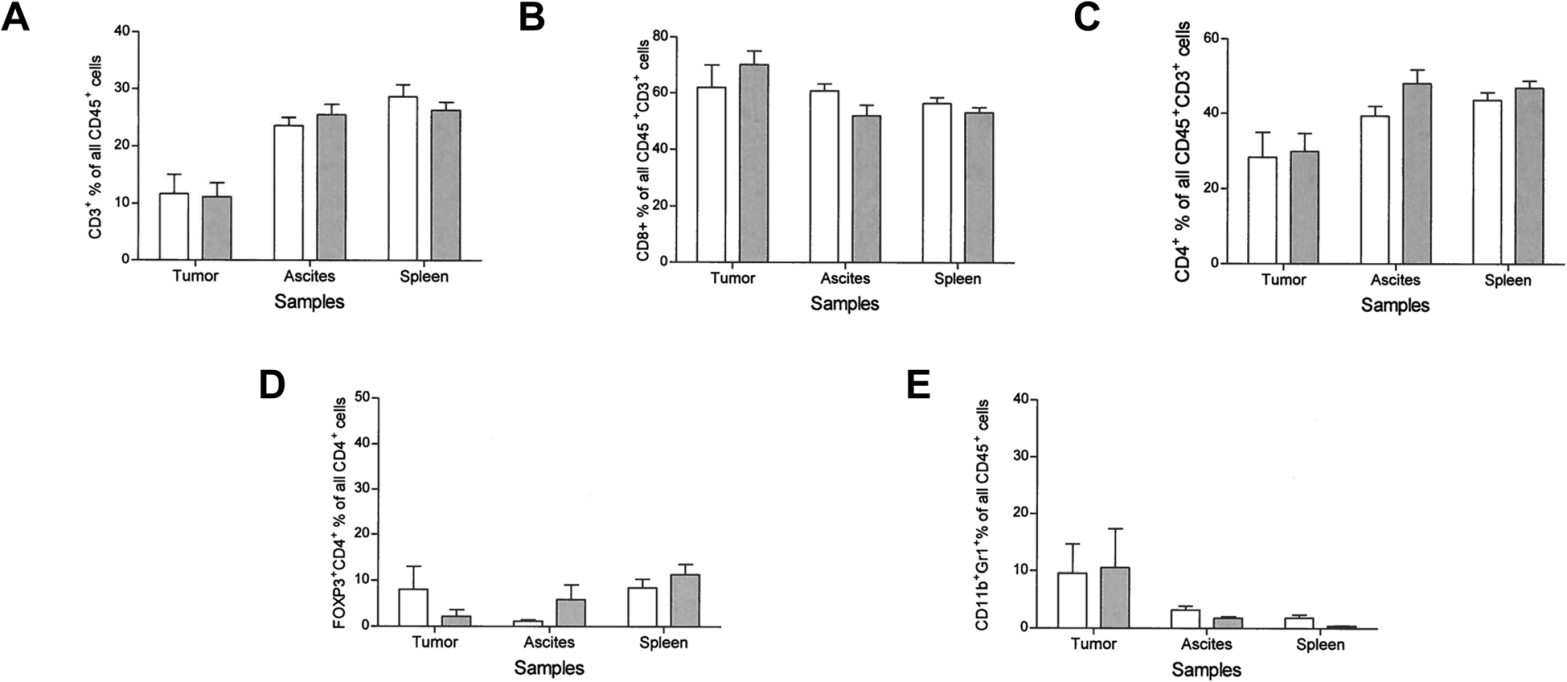
***Expression of codon-optimized firefly luciferase in ID8 cells does not alter cellular immune microenvironment*** **(A) CD3 T cells (y-axis) in ID8 Luc2 (Dark Gray, n = 6) compared to ID8 parental line (White bars, n = 8) in mouse tumor, ascites, and spleen (x-axis) at 12–15 weeks post tumor implant. (B)** CD8 T-cell populations, **(C)** CD4 T-Cell populations, **(D)** FOXP3 T-regulatory cells populations, **(E)** CD11bGr1 myeloid derived suppressor cells or MDSC’s. All cell subtypes were CD45^+^ gated. Horizontal bars are SEM.

The percentage of tumor infiltrating CD3+ CD45+ positive lymphocytes was not significantly different in the luc2 line compared to the parental line (p = 0.914; Figure 3A). There was also no significant differences seen in CD8+ populations infiltrating tumor (p = 0.4002) or in the CD4+ population (p = 0.8499; Figure 3B and C). The expression of luciferase also did not significantly alter the levels of regulatory T cells, FOXP3+ CD4+ cells (p = 0.3157; Figure 3D) or MDSC (p = 0.9108; Figure 3E) in tumors. There were no significant differences in these same cell populations in ascites or splenocytes (all p values >0.05) in mice implanted with ID8 luc2 tumors compared to the parental line, with the exception of an elevation in MDSC seen in the splenocytes of mice bearing the parental ID8 tumors compared to mice bearing the ID8 tumors expressing luc2 (p = 0.02).

### FOXP3+ T cells were more likely to be detected in the intraepithelial compartment and CD4+ T cells in the stroma as compared to CD3+ T cells, which were found equally in stroma and intraepithelial compartments

**Figure 4:**
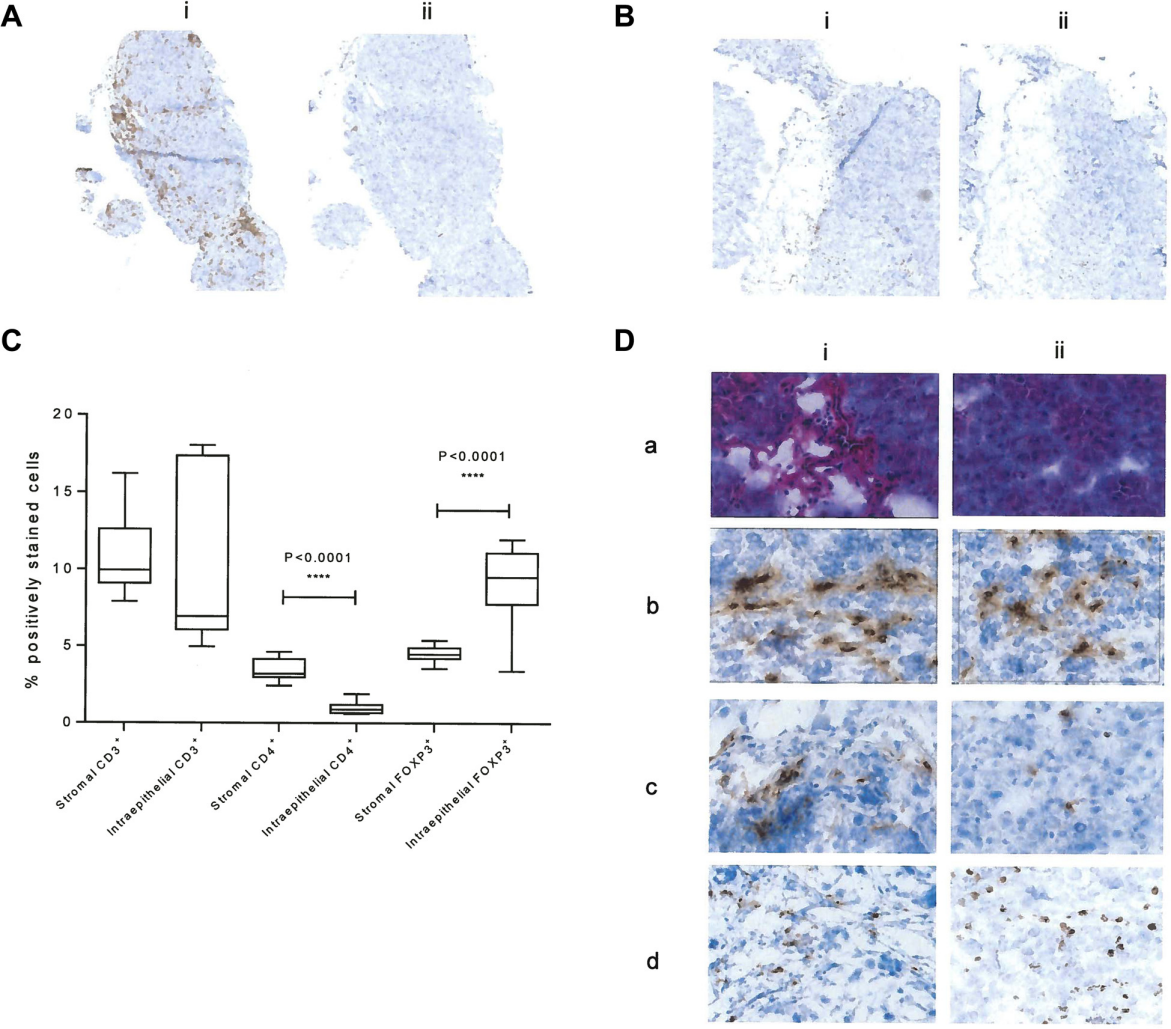
***FOXP3+ T cells were more likely to be detected in the intraepithelial compartment and CD4 T cells in the stroma compared to CD3 T cells, which were found equally in stroma and intraepithelial compartments*** **(A) CD3 immunohistochemistry (i) compared to Rat IgG isotype control (ii). (B)** FOXP3 T-cell immunohistochemistry (i) compared to Rat IgG isotype control (ii). **(C)** Box and whisker plot showing percent CD3 (n = 10), CD4 (n = 10), and FOXP3 (n = 10) cells from immunohistochemistry compared between stromal and intraepithelial sections. **(D)** Representative images of stromal and intraepithelial lymphocyte infiltration in the stromal (i) and intraepithelial (ii) compartments by H and E **(a)**, CD3 **(b)**, CD4 **(c)**, and FoxP3 **(d)**.

Immunohistochemical staining of ID8-luc2 tumors identified infiltration of CD3+ T cells (Figure 4A) and FOXP3+ cells (Figure 4B). No significant difference in percentage of positively stained CD3+ TILs was seen in the stroma compared to intraepithelial compartment. A significantly higher percentage of CD4+ TILs were detected in the stromal compartment compared to the intraepithelial compartment (p < 0.0001). Positively staining intraepithelial FOXP3+ were also significantly increased compared to stromal FOXP3+ (p < 0.0001; Figure 4C). Representative photomicrographs of tumor infiltrates are shown in Figure 4D.

## Discussion

Mouse models for ovarian cancer that can recapitulate the immune microenvironment will allow translational testing of immune based therapies. Ovarian cancer is an immunogenic tumor and the phenotype of the immune microenvironment has been shown to impact prognosis in a number of studies. The presence of CD3+ T cell infiltrates, and the homing of this infiltrate to the intraepithelial compartment, has been shown to be prognostic for improved survival by multivariate analysis [1-3,35]. Conversely, the development of immunomodulatory responses such as the recruitment of regulatory T cells and a low ratio of cytotoxic CD8+ to regulatory T cells has been shown to predict poor patient survival [2,8]. Systemic immune responses have also been shown to be important in survival [36].

Two common proteins used to visualize tumor cells *in vivo* are the green fluorescent protein (GFP) derived from the jellyfish *Aequorea victoria*, and luciferase derived from the firefly. Expression of foreign proteins in cancer lines must be done with caution especially in those cancers where knowledge of the immune interactions have been shown to effect clinical outcomes such as in ovarian cancer because expression of a xeno-protein may be immunogenic or alter the biology of the cancer. GFP expression in human cancer cells results in significant oxidative stress and can enhance cytotoxicity by upregulating total glutathione [37]. GFP has also been reported to promote caspase 3 activation and apoptosis [38]. Luciferase has been used successfully *in vivo* without negatively impacting tumor growth in immunocompetent mouse models of ovarian cancer [25,26]. We have shown that a codon-optimized version of this protein in mutant mice with an albino coat also propagates well *in vivo*. Codon-optimization and removal of cryptic splice sites has been shown to enhance the transmission of light and allows detection at the 10 cell level in albino mice [28].

Luciferase is known to be weakly immunogenic in Balb/c mice [39]. We have also shown immunogenicity of ID8 cells expressing luc2 in the C57Bl/6 immune background. In this model and this immune background, expression of luc2 does not alter the immune profile in tumor, ascites, or spleen in tumor bearing mice with the possible exception of MDSC, which is of uncertain significance since MDSC have been reported to change over time in this model [40]. Despite the stimulation of the immune system seen with the expression of luc2 in the transduced tumor line, engraftment rates and tumor growth did not differ.

The quantitation of CD3 T cells and CD4 T cells in the stromal and intraepithelial compartments also reflects what has been observed in human ovarian cancer. Sato and colleagues reported that CD3 T cells and CD4 T cells are found in higher numbers in the stromal compartments with increases in the means and medians of 5 to 6 fold and 8 to 9 fold for CD3 and CD4 respectively [2]. We saw the same trend in both CD3 and CD4 in when we quantitated stromal and intraepithelial staining of ID8 cells using the same techniques, although only CD4 was statistically significant in our more limited sample size. Although Sato and colleagues did not directly report stromal or intraepithelial FOXP3 staining, they attributed the negative survival impact of CD4 T cells to regulatory T cells. We see a significant increase in intraepithelial FOXP3 staining for the ID8 tumor compared to the stromal compartment, despite a decrease in CD4 staining suggesting enrichment of the regulatory T cells in the intraepithelial compartment. This is congruent with recognition of the importance of CD8 in the intraepithelial compartment in prognosis of human ovarian cancer.

## Conclusions

We conclude that use of a codon-optimized firefly luciferase expressed in ID8 cells in albino mice represents an immunocompetent model of ovarian cancer that maintains the tumor-host interactions seen without expression of the reporter gene. Humoral immune responses against a xeno-transgene do not correlate with tumor rejection and the tumor microenvironment is not altered. This system may allow effective preclinical testing of novel immune therapies and vaccines for ovarian cancer in a mouse model that replicates the immune microenvironment. The ability to detect and quantitate microscopic disease *in vivo* will also allow the study of strategies targeting optimally cytoreduced or clinical remissions to prevent recurrence.

## Additional files

Additional file 1: Figure S1.
*Selection of transduced luc2 tumor cell lines* Selection of ID8 cell lines transduced with luc2 and tested for expression by the addition of d-luciferin substrate *in vitro* compared to parental line. (A) was selected as a representative low expression line and (B) was selected as a representative high expression line. Each bar represents a single mean of 4 replicates for a single subline. Error bars represent standard error. Additional file 2: Figure S2.
*Comparison of mouse type, cell line, and cell load* (A) Total flux (Y-axis) versus time in weeks (x-axis) for each experimental group. Experimental groups do not differ significantly. (B) Serum antibody concentration (Y-axis) versus time in weeks (x-axis) for each experimental group. Circles denote Luc2 A clone, Squares: Luc2B clone. Filled in shapes represent a cell load of 1x10^6^ cells/mouse. Open shapes represent a cell load of 5x10^6^ cells/mouse. Four replicates per condition: 2 cell lines (A -low expression or B -high expression), 2 cell loads (1x10^6^ cells/mouse or 5x10^6^ cells/mouse), and 2 mouse types C57/BL/6/BrdCsHsd-Tyr^c^ (Harlan Laboratories) or B6(Cg)-Tyrc-2 J/J mice (Jackson Laboratories) Total mice: n = 32.

## References

[1] Zhang L, Conejo-Garcia JR, Katsaros D, Gimotty PA, Massobrio M, Regnani G (2003). Intratumoral T cells, recurrence, and survival in epithelial ovarian cancer. N Engl J Med.

[2] Sato E, Olson SH, Ahn J, Bundy B, Nishikawa H, Qian F (2005). Intraepithelial CD8+ tumor-infiltrating lymphocytes and a high CD8+/regulatory T cell ratio are associated with favorable prognosis in ovarian cancer. Proc Natl Acad Sci U S A.

[3] Adams SF, Levine DA, Cadungog MG, Hammond R, Facciabene A, Olvera N (2009). Intraepithelial T cells and tumor proliferation: impact on the benefit from surgical cytoreduction in advanced serous ovarian cancer. Cancer.

[4] Clarke B, Tinker AV, Lee CH, Subramanian S, van de Rijn M, Turbin D (2009). Intraepithelial T cells and prognosis in ovarian carcinoma: novel associations with stage, tumor type, and BRCA1 loss. Mod Pathol.

[5] Hamanishi J, Mandai M, Iwasaki M, Okazaki T, Tanaka Y, Yamaguchi K (2007). Programmed cell death 1 ligand 1 and tumor-infiltrating CD8+ T lymphocytes are prognostic factors of human ovarian cancer. Proc Natl Acad Sci U S A.

[6] Shah CA, Allison KH, Garcia RL, Gray HJ, Goff BA, Swisher EM (2008). Intratumoral T cells, tumor-associated macrophages, and regulatory T cells: association with p53 mutations, circulating tumor DNA and survival in women with ovarian cancer. Gynecol Oncol.

[7] Nielsen JS, Sahota RA, Milne K, Kost SE, Nesslinger NJ, Watson PH (2012). CD20+ tumor-infiltrating lymphocytes have an atypical CD27- memory phenotype and together with CD8+ T cells promote favorable prognosis in ovarian cancer. Clin Cancer Res.

[8] Curiel TJ, Coukos G, Zou L, Alvarez X, Cheng P, Mottram P (2004). Specific recruitment of regulatory T cells in ovarian carcinoma fosters immune privilege and predicts reduced survival. Nat Med.

[9] Winter WE, Maxwell GL, Tian C, Sundborg MJ, Rose GS, Rose PG (2008). Tumor residual after surgical cytoreduction in prediction of clinical outcome in stage IV epithelial ovarian cancer: a Gynecologic Oncology Group Study. J Clin Oncol.

[10] Kullander S, Rausing A, Trope C (1978). Human ovarian tumours heterotransplanted to "nude" mice. Acta Obstet Gynecol Scand.

[11] Yokota SJ, Facciponte JG, Kelleher RJ, Shultz LD, Loyall JL, Parsons RR (2013). Changes in ovarian tumor cell number, tumor vasculature, and T cell function monitored in vivo using a novel xenograft model. Cancer Immun.

[12] Bankert RB, Balu-Iyer SV, Odunsi K, Shultz LD, Kelleher RJ, Barnas JL (2011). Humanized mouse model of ovarian cancer recapitulates patient solid tumor progression, ascites formation, and metastasis. PLoS One.

[13] Geller MA, Knorr DA, Hermanson DA, Pribyl L, Bendzick L, McCullar V (2013). Intraperitoneal delivery of human natural killer cells for treatment of ovarian cancer in a mouse xenograft model. Cytotherapy.

[14] Dinulescu DM, Ince TA, Quade BJ, Shafer SA, Crowley D, Jacks T (2005). Role of K-ras and Pten in the development of mouse models of endometriosis and endometrioid ovarian cancer. Nat Med.

[15] Orsulic S, Li Y, Soslow RA, Vitale-Cross LA, Gutkind JS, Varmus HE (2002). Induction of ovarian cancer by defined multiple genetic changes in a mouse model system. Cancer Cell.

[16] Kim J, Coffey DM, Creighton CJ, Yu Z, Hawkins SM, Matzuk MM (2012). High-grade serous ovarian cancer arises from fallopian tube in a mouse model. Proc Natl Acad Sci U S A.

[17] Connolly DC, Bao R, Nikitin AY, Stephens KC, Poole TW, Hua X (2003). Female mice chimeric for expression of the simian virus 40 TAg under control of the MISIIR promoter develop epithelial ovarian cancer. Cancer Res.

[18] El-Naggar SM, Malik MT, Martin A, Moore JP, Proctor M, Hamid T (2007). Development of cystic glandular hyperplasia of the endometrium in Mullerian inhibitory substance type II receptor-pituitary tumor transforming gene transgenic mice. J Endocrinol.

[19] Liang S, Yang N, Pan Y, Deng S, Lin X, Yang X (2009). Expression of activated PIK3CA in ovarian surface epithelium results in hyperplasia but not tumor formation. PLoS One.

[20] Roberts PC, Mottillo EP, Baxa AC, Heng HH, Doyon-Reale N, Gregoire L (2005). Sequential molecular and cellular events during neoplastic progression: a mouse syngeneic ovarian cancer model. Neoplasia.

[21] Cancer Genome Atlas Research Network. Integrated genomic analyses of ovarian carcinoma. Nature. 2011;474(7353):609–15. doi:10.1038/nature10166. Erratum in: Nature. 2012 Oct 11;490(7419):298. PubMed PMID: 21720365; PubMed Central PMCID: PMC3163504. 10.1038/nature10166 PMC3163504 21720365

[22] Jacobs AJ, Curtis GL, Newland JR, Wilson RB, Ryan WL (1984). Chemical induction of ovarian epithelial carcinoma in mice. Gynecol Oncol.

[23] Nishida T, Sugiyama T, Kataoka A, Ushijima K, Yakushiji M (1998). Histologic characterization of rat ovarian carcinoma induced by intraovarian insertion of a 7,12-dimethylbenz[a]anthracene-coated suture: common epithelial tumors of the ovary in rats?. Cancer.

[24] Roby KF, Taylor CC, Sweetwood JP, Cheng Y, Pace JL, Tawfik O (2000). Development of a syngeneic mouse model for events related to ovarian cancer. Carcinogenesis.

[25] Hung CF, Tsai YC, He L, Wu TC (2007). Control of mesothelin-expressing ovarian cancer using adoptive transfer of mesothelin peptide-specific CD8+ T cells. Gene Ther.

[26] Toyoshima M, Tanaka Y, Matumoto M, Yamazaki M, Nagase S, Sugamura K (2009). Generation of a syngeneic mouse model to study the intraperitoneal dissemination of ovarian cancer with in vivo luciferase imaging. Luminescence.

[27] Li C, Chacko AM, Hu J, Hasegawa K, Swails J, Grasso L (2014). Antibody-based tumor vascular theranostics targeting endosialin/TEM1 in a new mouse tumor vascular model. Cancer Biol Ther.

[28] Rabinovich BA, Ye Y, Etto T, Chen JQ, Levitsky HI, Overwijk WW (2008). Visualizing fewer than 10 mouse T cells with an enhanced firefly luciferase in immunocompetent mouse models of cancer. Proc Natl Acad Sci U S A.

[29] Neff BA, Voss SG, Allen C, Schroeder MA, Driscoll CL, Link MJ (2009). Bioluminescent imaging of intracranial vestibular schwannoma xenografts in NOD/SCID mice. Otol Neurotol.

[30] Goodell V, McNeel D, Disis ML (2008). His-tag ELISA for the detection of humoral tumor-specific immunity. BMC Immunol.

[31] Lu H, Knutson KL, Gad E, Disis ML (2006). The tumor antigen repertoire identified in tumor-bearing neu transgenic mice predicts human tumor antigens. Cancer Res.

[32] Park KH, Gad E, Goodell V, Dang Y, Wild T, Higgins D (2008). Insulin-like growth factor-binding protein-2 is a target for the immunomodulation of breast cancer. Cancer Res.

[33] Dang Y, Wagner WM, Gad E, Rastetter L, Berger CM, Holt GE (2012). Dendritic cell-activating vaccine adjuvants differ in the ability to elicit antitumor immunity due to an adjuvant-specific induction of immunosuppressive cells. Clin Cancer Res.

[34] Goodell V, Salazar LG, Urban N, Drescher CW, Gray H, Swensen RE (2006). Antibody immunity to the p53 oncogenic protein is a prognostic indicator in ovarian cancer. J Clin Oncol.

[35] Hamanishi J, Mandai M, Iwasaki M, Okazaki T, Tanaka Y, Yamaguchi K (2007). Programmed cell death 1 ligand 1 and tumor-infiltrating CD8+ T lymphocytes are prognostic factors of human ovarian cancer. Proc Natl Acad Sci U S A.

[36] Milne K, Barnes RO, Girardin A, Mawer MA, Nesslinger NJ, Ng A (2008). Tumor-infiltrating T cells correlate with NY-ESO-1-specific autoantibodies in ovarian cancer. PLoS One.

[37] Goto H, Yang B, Petersen D, Pepper KA, Alfaro PA, Kohn DB (2003). Transduction of green fluorescent protein increased oxidative stress and enhanced sensitivity to cytotoxic drugs in neuroblastoma cell lines. Mol Cancer Ther.

[38] Liu HS, Jan MS, Chou CK, Chen PH, Ke NJ (1999). Is green fluorescent protein toxic to the living cells?. Biochem Biophys Res Commun.

[39] Petkov SP, Heuts F, Krotova OA, Kilpelainen A, Engstrom G, Starodubova ES (2013). Evaluation of immunogen delivery by DNA immunization using non-invasive bioluminescence imaging. Hum Vaccin Immunother.

[40] Duraiswamy J, Freeman GJ, Coukos G (2013). Therapeutic PD-1 pathway blockade augments with other modalities of immunotherapy T-cell function to prevent immune decline in ovarian cancer. Cancer Res.

