# Supplementary figures and images for: Preservation of tumor-host immune interactions with luciferase-tagged imaging in a murine model of ovarian cancer
Source: J Immunother Cancer. 2015 May 19;3:16. doi: 10.1186/s40425-015-0060-6 (PMC4437454; doi:10.1186/s40425-015-0060-6)

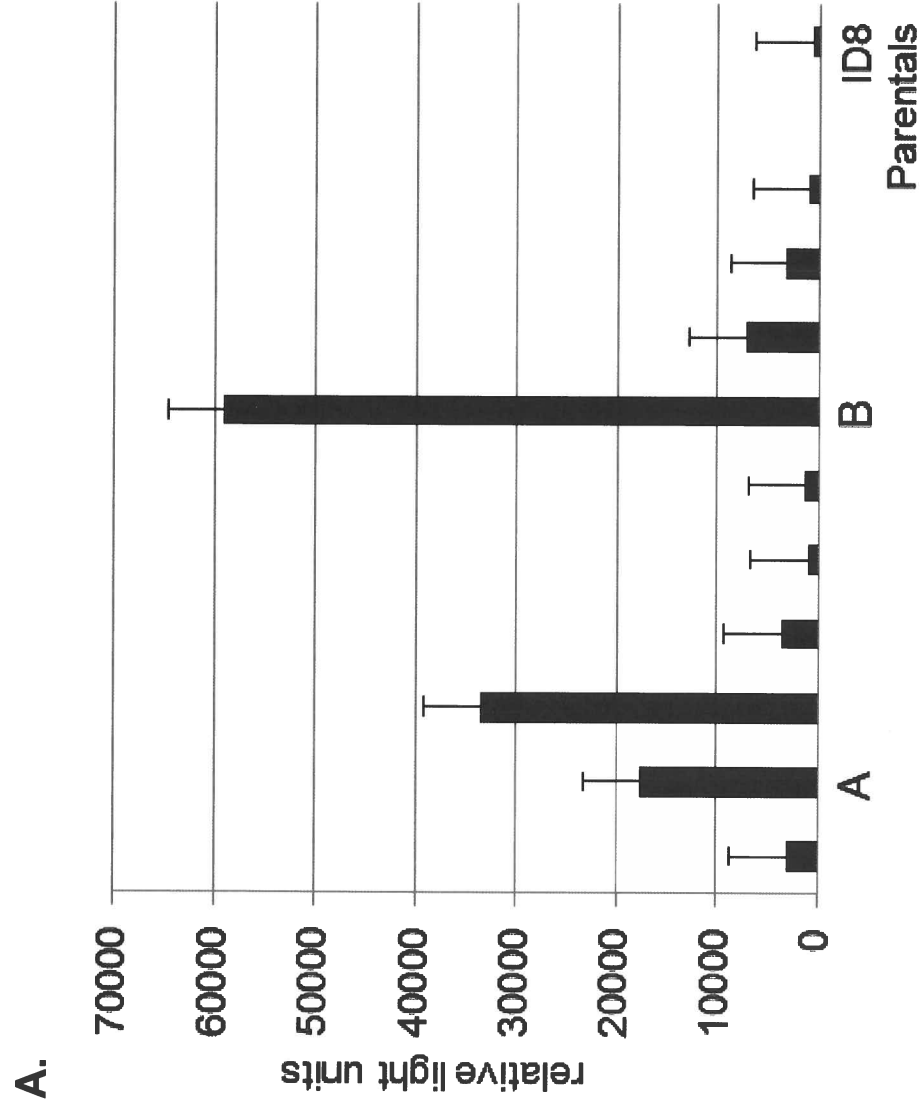

Supplement: Additional file 1: Figure S1. — Selection of transduced luc2 tumor cell lines Selection of ID8 cell lines transduced with luc2 and tested for expression by the addition of d-luciferin substrate in vitro compared to parental line. (A) was selected as a representative low expression line and (B) was selected as a representative high expression line. Each bar represents a single mean of 4 replicates for a single subline. Error bars represent standard error. [file 40425_2015_60_MOESM1_ESM.pdf]

**A.**

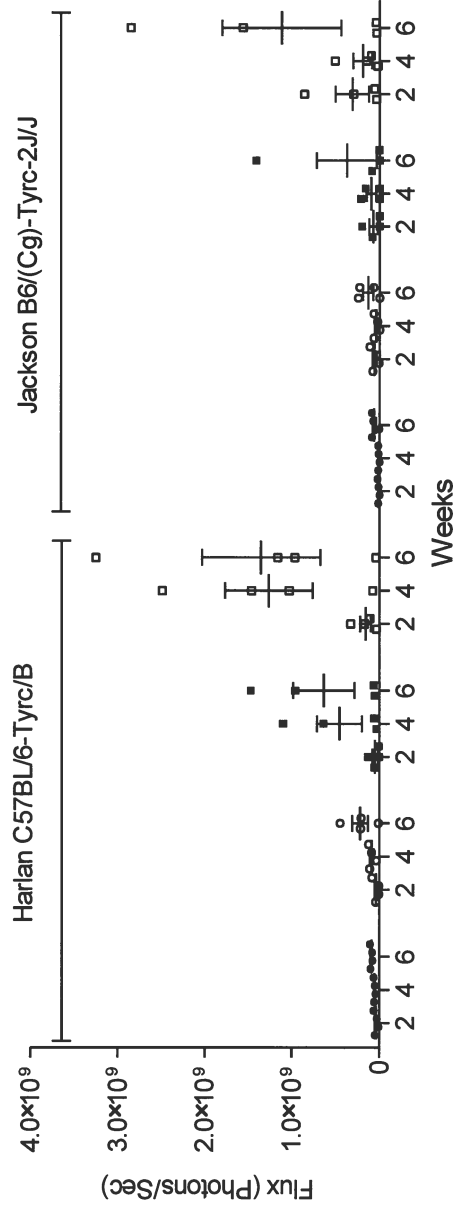

**B.**

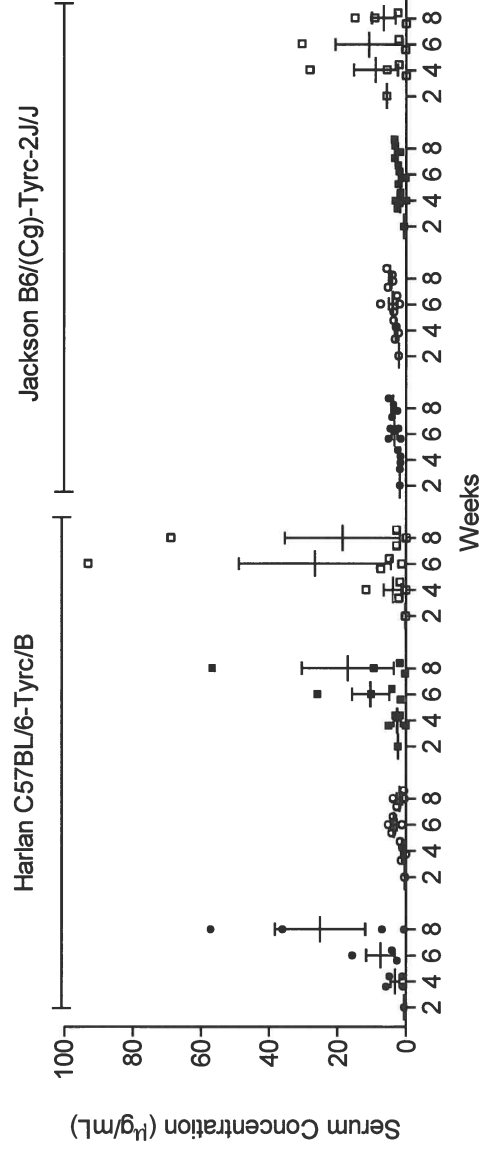

Supplement: Additional file 2: Figure S2. — Comparison of mouse type, cell line, and cell load (A) Total flux (Y-axis) versus time in weeks (x-axis) for each experimental group. Experimental groups do not differ significantly. (B) Serum antibody concentration (Y-axis) versus time in weeks (x-axis) for each experimental group. Circles denote Luc2 A clone, Squares: Luc2B clone. Filled in shapes represent a cell load of 1x10^6^ cells/mouse. Open shapes represent a cell load of 5x10^6^ cells/mouse. Four replicates per condition: 2 cell lines (A -low expression or B -high expression), 2 cell loads (1x10^6^ cells/mouse or 5x10^6^ cells/mouse), and 2 mouse types C57/BL/6/BrdCsHsd-Tyrc (Harlan Laboratories) or B6(Cg)-Tyrc-2 J/J mice (Jackson Laboratories) Total mice: n = 32. [file 40425_2015_60_MOESM2_ESM.pdf]
